# Comparison of BD MAX GBS and GenomEra GBS assays for rapid intrapartum PCR detection of vaginal carriage of group B streptococci

**DOI:** 10.1371/journal.pone.0215314

**Published:** 2019-04-16

**Authors:** Trine Andreasen, Jens Kjølseth Møller, Mohammed Rohi Khalil

**Affiliations:** 1 Department of Clinical Microbiology, Lillebaelt Hospital, Vejle, Denmark; 2 Institute of Regional Health Research, Faculty of Health Sciences, University of Southern Denmark, Odense, Denmark; 3 Department of Gynecology and Obstetrics, Lillebaelt Hospital, Kolding, Denmark; All India Institute of Medical Sciences, INDIA

## Abstract

**Objective:**

To compare the diagnostic performance of BD MAX and GenomEra PCR assays for a rapid PCR detection of vaginal carriage of group B streptococci at delivery.

**Methods:**

This is a retrospective laboratory analysis of vaginal swab samples taken intrapartum from a randomly selected cohort of pregnant women giving birth at a single childbirth and maternity unit.

**Results:**

Ninety-one culture-positive and 279 culture-negative vaginal samples were included from a cohort of 902 women. One-hundred-and-two specimens were found positive with the BD MAX and 84 with the GenomEra PCR assay. No statistically significant difference was observed compared to culture, sensitivity of BD MAX 84.6% (77/91) [95%CI 75.5–91.3] and of GenomEra 71.4% (65/91) [95%CI 61.0–80.4]. When compared to a combined reference standard, no statistically significant differences were seen between culture, BD MAX and GenomEra PCR assays. The sensitivities were 82.7% (91/110) [95%CI 74.3–89.3], 87.3% (96/110) [95%CI 79.6–92.9], and 79.1% (87/110) [95%CI 70.3–86.3], respectively.

**Conclusion:**

Both PCR assays performed comparably to culture of the intrapartum vaginal samples. In particular, the GenomEra assay is potentially an easy and rapid on-site PCR test for intrapartum detection of vaginal carriage of group B streptococci at a maternity ward to identify women who should receive intrapartum antibiotic prophylaxis.

## Introduction

Group B streptococci (GBS) are the most frequent cause of early-onset neonatal infection, which is associated with significant morbidity and mortality among infants. The incidence rate of early-onset GBS infection ranges from 0.5 to 3.0 per 1,000 live births, with 4–10% mortality [1–4]. In their guidelines, Centers for Disease Control and Prevention (CDC) endorse universal culture-based antenatal screening for GBS colonization in all pregnant women between 35 and 37 weeks of gestation to identify women who should receive intrapartum antibiotic prophylaxis [1, 2]. However, a national cohort study found that samples for culture screening between week 35 and 37 of gestation were negative for 81% of the mothers of babies who developed early-onset neonatal group B streptococcal disease [5]. These data suggest that a change in colonization status may have occurred at the time of birth implying that antepartum sampling and culture are not optimal methods to provide a relevant GBS colonization status at delivery, resulting in missed opportunities to avoid GBS transmission from mother to infant during birth.

A rapid nucleic acid amplification test performed at the time of delivery may constitute an alternative screening method. Previous studies suggest that the polymerase chain reaction (PCR) test at delivery may accurately reflect intrapartum GBS colonization status [6–8]. A European consensus conference on intrapartum GBS screening and antibiotic prophylaxis recommends intrapartum antimicrobial prophylaxis based on a universal intrapartum GBS screening strategy using a rapid real-time testing [9]. However, a time-consuming broth enrichment step precludes the use of rapid on-site PCR detection of GBS carriage at the time of delivery.

The aim of this study was to compare an intrapartum culture for GBS with the diagnostic performance of BD MAX and GenomEra PCR assays without initial broth enrichment to achieve rapid PCR detection of vaginal carriage of group B streptococci at delivery and identify women who should receive intrapartum antibiotic prophylaxis.

## Material and methods

### Ethical approval

The clinical study was approved by the Regional Scientific Ethical Committees for Southern Denmark (S-20130089) and the Danish Data Protection Agency (2008-58-0035). All participants provided written informed consent.

### Study design

This study was designed to compare new GenomEra GBS PCR results on the same set of samples as previously obtained BD MAX GBS PCR results and culture of vaginal swab samples [10]. Originally, for practical reasons, culture was done on fresh samples and BD MAX GBS PCR on the same samples after one freeze-thaw cycle. Subsequently, GenomEra GBS PCR was performed on the frozen samples with an additional freeze-thaw cycle. Professional, fully qualified laboratory technicians at the Department of Clinical Microbiology performed culture, BD MAX PCR and GenomEra PCR assays. All sample aliquots used throughout the study, per included woman, came from the same ESwab sample tube. The culture results were blinded for the laboratory technicians prior to PCR analysis. In a small number of cases for both assays, the specimens were initially undetermined because of inhibition, reagent failure or system errors, which led to additional testing of the sample and repeating the DNA extraction and PCR assay. The results from both tests were interpreted and produced by the respective PCR equipment software as a qualitative response, either positive or negative for GBS. The results of the GBS culture, BD MAX GBS and GenomEra GBS assays were read and recorded separately by independent laboratory technicians. Discrepant PCR results between BD MAX and GenomEra were examined by a repeat PCR test of samples with both assays.

### Study population and sample collection

From a previous prospective observational study including 902 pregnant women giving birth at Lillebaelt Hospital between 2013 and 2014 [10], 91 culture positive and 279 culture negative intrapartum vaginal swab samples kept at -80°C were randomly selected and used to assess the performance of the GenomEra GBS PCR assay. The GenomEra results were compared with results from the original vaginal intrapartum culture and BD MAX GBS.

The collection of original specimens was as described by Khalil et al. 2017: Briefly, during labor, the midwife obtained one vaginal ESwab (Copan Diagnostics, Brescia, Italy) sample for both culture (reference standard) and PCR assays for GBS. All samples were cultured immediately as described below at the Department of Clinical Microbiology, Lillebaelt Hospital, Vejle, Denmark, and the specimen tubes containing the vaginal intrapartum ESwab sample medium were, for practical reasons, subsequently frozen at -80°C for later PCR analyses. The PCR analyses were performed as direct tests without initial enrichment of the specimens in a culture broth in order to mimic a rapid on-site intrapartum PCR test.

### Culture of original specimens

Direct plating was carried out by streaking the ESwab specimen on a selective Granada agar plate (BioMérieux, Spain). The Granada agar plates were incubated immediately after seeding in CO_2_-enriched atmosphere at 35°C. The Granada agar plates were read after one and two days of incubation. All GBS-like colonies (identified by their orange color) were routinely confirmed as *Streptococcus agalactiae* (GBS). The colonies were identified using the Microflex LT MALDI-TOF system (Bruker Daltonik, Germany). One colony from GBS positive cultures was dispersed into 1 mL of broth medium supplemented with 10% glycerol (Statens Serum Institut, Denmark) and stored in a -80-degree freezer.

### BD MAX real-time GBS PCR

The BD MAX System (Becton Dickinson, USA) automatically extracts the nucleic acid using a combination of heat, lytic enzymes and magnetic capture beads. The BD MAX GBS PCR assay (Cat. No. 441772) amplifies a section of the *cfb*-gene target sequence of the GBS chromosome. The assay has previously been evaluated against culture for GBS on clinical specimens from routine prenatal screening of women in USA [11]. The BD MAX assay includes an Internal Process Control to monitor for the presence of potential inhibitory substances as well as system or reagent failures that may occur during the process. A sample volume of 300 μL was determined empirically by the laboratory of the Department of Clinical Microbiology as the optimal volume for GBS detection. The assay run takes approximately 120 minutes including reporting of results. Results were interpreted according to the manufacturer’s instructions.

### GenomEra GBS PCR assay

The GenomEra CDX system (Abacus Diagnostica, Finland) is a molecular diagnostic analyzer consisting of an integrated thermal cycler and a time-resolved fluorometer. The GenomEra GBS PCR kit targets an internal region of the *cfb*-gene and, based on in-silico analysis of published GBS genomes and experimental data on a selection of GBS strains, is expected to detect all clinical GBS isolates (personal contact with Abacus Diagnostica, 2018). The GenomEra GBS PCR assay is clinically validated and CE-IVD-marked for use only with pre-enrichment broth culture of samples (GenomEra package insert).

All the reagents required to perform the amplification and detection steps are readily contained in dry form in the Test Chips, including an Internal Amplification Control (IAC) of a non-naturally occurring DNA sequence to monitor for assay inhibition. The GenomEra GBS assay kit also includes Z-tubes containing zirconium particles for specimen dilution and cell disruption. In this study, we modified the manufacturer’s instructions as we applied direct swab samples instead of a 4-hour pre-enrichment broth culture of samples, and a sample volume of 60 μl of ESwab medium instead of 10 μl of enrichment culture medium. The volume was increased in order to achieve a sample volume more comparable to the one used in the BD MAX assay. Abacus Diagnostica recommended the change to 60 μl to maximize the sensitivity of the GenomEra assay and concomitantly ensure that the Test Chips were not overloaded, as overloading would produce invalid results. The 60 μl of ESwab medium was added to 1,000 μl of the GenomEra buffer supplied for swab samples. Samples were lysed by vortexing for five minutes. From this mixture, 35 μl was transferred to the Test Chip and analyzed on the GenomEra CDX system. The assay takes approximately 60 minutes including the final reporting of results. Results were interpreted according to the manufacturer’s instructions.

### Combined reference standard

Discrepant PCR results on samples were examined by repeat testing of samples with both assays so the BD MAX and GenomEra assays were given equal opportunity. A combined reference standard was established by defining true positives as either culture positive samples or culture negative samples where one or more PCR test results (initial or repeat tests) with both the BD MAX GBS and the GenomEra GBS PCR assays were positive.

### Statistics

StataCorp. 2015. Stata Statistical Software: Release 14. College Station, TX: StataCorp LP was used for the statistical analysis.

## Results

### Performance of the BD MAX GBS and the GenomEra GBS assays with culture as reference

Initial (first run of) PCR tests resulted in 102 positive samples with the BD MAX GBS and 84 with the GenomEra GBS assay (Table 1). Seventy-seven (85%) of the culture-positive specimens were positive with BD MAX and 65 (71%) with GenomEra. Twenty-five of the 279 culture-negative samples were BD MAX GBS-positive, thus a specificity of about 91% was achieved (Table 2). The corresponding figures for the GenomEra GBS assay were 19 PCR positives of the 279 culture negative samples, and a specificity of about 93% (Table 2). Differences in sensitivity, specificity and predictive values between BD MAX and GenomEra with vaginal culture as the reference standard were not statistically significant (Table 2).

**Table 1:** First run PCR results with culture as reference.

Culture (fresh sample)	BD MAX 1^st^ run (frozen sample)	GenomEra 1^st^ run (frozen sample)	Number
**+**	**+**	**+**	65
**+**	**+**	**-**	12
**+**	**-**	**-**	14
**-**	**+**	**+**	18
**-**	**+**	**-**	7
**-**	**-**	**+**	1
**-**	**-**	**-**	253

Patterns of results of 370 intrapartum collected vaginal ESwab specimens examined by culture, BD MAX GBS and GenomEra GBS PCR.

+ = GBS positive, − = GBS negative.

**Table 2:** Comparison of the performance characteristics.

	BD MAX (1^st^ run)		GenomEra (1^st^ run)	
	% (n/N)	(95% CI)	% (n/N)	(95% CI)
Sensitivity	84.6 (77/91)	75.5–91.3	71.4 (65/91)	61.0–80.4
Specificity	91.0 (254/279)	87.1–94.1	93.2 (260/279)	89.6–95.9
PPV	75.5 (77/102)	66.0–83.5	77.4 (65/84)	67.0–85.8
NPV	94.8 (254/268)	91.4–97.1	90.9 (260/286)	87.0–94.0

Statistical analysis of the BD MAX and GenomEra GBS assay results with the intrapartum vaginal culture result as the reference standard.

CI = confidence interval, PPV = positive predictive value, NPV = negative predictive value

### Discrepancy analysis

Twenty of the total 370 specimens gave discordant results between BD MAX and GenomEra (Table 3). Eight of these 20 specimens were culture-negative and 12 culture-positive. All 20 specimens were re-tested (second run) with both BD MAX and GenomEra. One sample was lost for follow-up due to limited sample volume. For several of the specimens, repeat testing yielded a different result than that obtained in the first round (Table 3). Notably, seven of the eight culture-negative specimens were initially positive with BD MAX, while only one was positive with GenomEra. Four samples continued to be positive in repeat testing with BD MAX, while one specimen changed status from positive to negative with GenomEra. Six initially BD MAX-positive samples were negative in a repeat test with BD MAX, while six samples changed status from negative to positive with GenomEra.

**Table 3:** Divergent results between BD MAX and GenomEra.

Sample no.	Culture	BD MAX		GenomEra		Combined reference standard
	Result	1^st^ run	2^nd^ run	1^st^ run	2^nd^ run	
1	*Neg*	*Neg*	*Neg*	Pos	*Neg*	*Neg*
2	*Neg*	Pos	Pos	*Neg*	*Neg*	*Neg*
3	*Neg*	Pos	Pos	*Neg*	*Neg*	*Neg*
4	*Neg*	Pos	*Neg*	*Neg*	*Neg*	*Neg*
5	*Neg*	Pos	*Neg*	*Neg*	*Neg*	*Neg*
6	*Neg*	Pos	Pos	*Neg*	*Neg*	*Neg*
7	*Neg*	Pos	*Neg*	*Neg*	Borderline	*Neg*
8	*Neg*	Pos	Pos	*Neg*	Pos	Pos
9	Pos (3)	Pos	*Pos*	*Neg*	Pos	Pos
10	Pos (3)	Pos	Inhibition	*Neg*	Failed!	Pos
11	Pos (3)	Pos	*Neg*	*Neg*	*Neg*	Pos
12	Pos (2)	Pos	Pos	*Neg*	*Neg*	Pos
13	Pos (3)	Pos	Pos	*Neg*	Pos	Pos
14	Pos (NR)	Pos	*Neg*	*Neg*	Pos	Pos
15	Pos (3)	Pos	*Neg*	*Neg*	Pos	Pos
16	Pos (2)	Pos	*Neg*	*Neg*	*Neg*	Pos
17	Pos (2)	Pos	Pos	*Neg*	Pos	Pos
18	Pos (3)	Pos	ND	*Neg*	ND	Pos
19	Pos (2)	Pos	*Neg*	*Neg*	*Neg*	Pos
20	Pos (3)	Pos	*Neg*	*Neg*	Pos	Pos

Re-test results of vaginal intrapartum specimens with discordant first run results by BD MAX and GenomEra. Culture results and the combined reference standard are also listed.

Neg = negative, Pos = positive, ND = not determined because of limited sample material.

The cultures were classified by semi-quantitative growth evaluation as having growth of only a few (1), some (2) or many (3) GBS colonies in intrapartum vaginal culture. NR = quantitative culture assessment not registered.

GBS isolates frozen from the 12 original culture-positive samples that were GenomEra-negative were re-cultured, re-identified as GBS by MALDI-TOF mass spectrometry, and tested using the GenomEra system to establish whether the original PCR-negative results were due to the lack of the target for the GenomEra PCR assay within the GBS strains cultured. All 12 samples tested positive with the GenomEra PCR assay, indicating that the original negative PCR results on the clinical samples were probably due to poor quality GBS DNA in the frozen clinical samples.

### Performance of the BD MAX GBS and the GenomEra GBS assays with a combined standard as reference

Using the combined reference standard, 110 of the 370 samples in the study were classified as true positives. These comprised 91 culture-positive samples, 18 culture-negative samples where both PCR assays were initially positive, and one culture-negative sample, for which both PCR assays had one or more positive test results in initial or repeat assays, respectively. Distributions of results are shown in Table 4.

**Table 4:** PCR results with combined standard as reference.

Combined reference standard	Culture	BD MAX	GenomEra	Number
**+**	**+**	**+**	**+**	65
**+**	**+**	**+**	**(+)**	3
**+**	**+**	**+**	**-**	9
**+**	**+**	**-**	**-**	14
**+**	**-**	**+**	**+**	18
**+**	**-**	**+**	**(+)**	1
**-**	**-**	**+**	**-**	6
**-**	**-**	**-**	**+**	1
**-**	**-**	**-**	**-**	253

BD MAX and GenomEra GBS test results compared to the combined reference standard.

(+) repeat test positive.

Table 5 shows the performance of cultures of GBS, BD MAX GBS and GenomEra GBS compared to the combined reference standard. Differences in sensitivity, specificity and predictive values between vaginal culture, BD MAX GBS and GenomEra GBS were not statistically significant.

**Table 5:** Performance characteristics of culture and PCR assays with the combined standard as reference.

	Culture		BD MAX		GenomEra	
	% (n/N)	(95% CI)	% (n/N)	(95% CI)	% (n/N)	(95% CI)
Sensitivity	82.7 (91/110)	74.3–89.3	87.3 (96/110)	79.6–92.9	79.1 (87/110)	70.3–86.3
Specificity	100 (260/260)	98.6–100	97.7 (254/260)	95.0–99.1	99.6 (259/260)	97.9–100
PPV	100 (91/91)	96.0–100	94.1 (96/102)	87.6–97.8	98.9 (87/88)	93.8–100
NPV	93.2 (260/279)	89.6–95.9	94.8 (254/268)	91.4–97.1	91.8 (259/282)	88.0–94.8

Comparison of the statistical analysis of the culture, BD MAX GBS, and GenomEra GBS assay results compared with the combined reference standard.

CI = confidence interval, PPV = positive predictive value, NPV = negative predictive value

## Discussion

This study was designed to compare the diagnostic accuracy of the BD MAX GBS assay and a new GenomEra GBS assay for rapid intrapartum PCR detection of vaginal carriage of group B streptococci using direct sample material from vaginal swabs. Based on a direct comparison with culture, BD MAX GBS had slightly better sensitivity, but lower specificity compared to GenomEra GBS, although the differences in performance were not statistically significant. Both PCR assays failed to detect GBS in all culture-positive vaginal samples, although they did detect GBS in several culture-negative specimens. Compared to a defined combined reference standard, there were no statistically significant differences between the respective performance characteristics of culture, BD MAX GBS PCR and GenomEra GBS PCR. The sensitivities of the three assays for detection of GBS in intrapartum vaginal ESwab samples were 83.0%, 87.3%, and 79.1%, respectively. Similar sensitivities were seen in a smaller study examining intrapartum GBS colonization status using GenomEra GBS PCR assay compared to culture [12].

The strength of our study is that the vaginal swab samples used are randomly chosen from a large cohort of prospectively sampled women giving birth at Lillebaelt Hospital [10]. Furthermore, we employed the GBS PCR assays without broth pre-enrichment steps prior to the PCR analyses in order to create a realistic screening scenario for intrapartum testing during labor, since enrichment cultures preclude the practical use of intrapartum PCR assays for detection of GBS in women giving birth.

Discrepancies between the results of culture and the two PCR assays on the clinical sample material may indicate true differences in clinical sensitivity and specificity but could also be due to the limitations of the study design and differences in the PCR protocols. By employing a combined reference standard, three repeatedly BD MAX positive samples are regarded as false positives. On the other hand, six of twelve initially BD MAX positive and culture positive samples were not confirmed positive by a repeat PCR test. In contrast, seven of twelve initially GenomEra-negative and culture-positive samples were positive by the repeat test. One weakness of our study is that, for practical reasons, the two PCR assays were compared based on frozen sample material rather than concomitantly with the culture of fresh samples for GBS. This may explain the PCR-negative results for both the BD MAX and GenomEra assay on 14 culture-positive samples, a conjecture underpinned by the fact that the majority of culture-positive samples showed growth of only a few GBS colonies. This may have led to a low number of available targets and less scope for a positive result between the first and second tests of independently prepared samples of a specimen. Notably, the discrepancy analysis seems to indicate that both PCR assays were affected by freeze-thawing because some of the BD MAX samples that were initially positive, changed status to negative despite a high number of GBS-colony-forming units in the samples, based on the semi-quantitative culture results. It is also relevant to note that, in a recent clinical study using fresh vaginal-rectal swab specimens, the BD MAX GBS PCR assay detected 61 specimens with and 62 without enrichment culture, respectively, among 62 culture-positive specimens[13].

Another weakness is that the GenomEra PCR assay was applied after an additional freeze-thaw cycle of the clinical samples. It could be expected that the sensitivity of the GenomEra GBS PCR assay is higher when the test is performed on fresh specimens.

It could be argued that the volume of sample material used in the PCR assays is partial to the BD MAX GBS assay, especially in samples with low GBS DNA load or poor DNA integrity. Among positive culture samples with low GBS colony numbers, a positive PCR result may be less likely due to the limited sample volumes applied in PCR assays [14]. BD MAX GBS PCR includes concentrating sample preparation with magnetic capture beads and thus all the original 300 μl of GBS sample material may end up in the amplification reaction. By way of contrast, in the GenomEra assay, the original sample of 60 μl is diluted in 1,000 μl of buffer, and 35 μl of this dilution is then used for PCR. The volume of sample material used in BD MAX may be higher by order of magnitude, even if the sample volume used with the GenomEra assay is increased from 10 to 60 μl. However, before the clinical study, the utilization of frozen specimens and an altered volume was not validated on clinical samples material or reference strains of GBS.

The direct and rapid use of BD MAX GBS is clearly a limitation because 3.4% of all specimens were initially undetermined due to either inhibition of the amplification of the Internal Process Control or technical failure of the PCR test. For specimens tested by GenomEra, the corresponding figure was only 2.0%.

Omitting LIM broth enrichment of the specimen before a PCR assay for vaginal GBS detection implies a small but statistically significant reduction in sensitivity (92.7% versus 99.1%), compared to the use of the same PCR test with prior LIM broth inoculation [15]. However, an LIM broth enrichment step 18 hours prior to the PCR assay precludes the use of a rapid on-site GBS PCR test during birth. Screening women for GBS during pregnancy at week 35–37 is known to fail to identify a large number of women with intrapartum carriage of GBS [7, 16, 17]. Thus, direct, rapid intrapartum GBS PCR screening seems preferable to antepartum GBS screening by culture or PCR with prior LIM broth enrichment of the specimens.

Several studies have confirmed that intrapartum PCR tests detect GBS more accurately than conventional GBS culture methods [7, 8, 14, 16, 18–20]. Screening of both vaginal and rectal specimens for GBS increases the yield of GBS-positive antepartum culture samples and the predictive values for intrapartum vaginal colonization status [9, 10]. Simultaneous intrapartum sampling of vagina and rectum for a rapid PCR assay could increase the yield of detection at birth, as the gastrointestinal tract is the natural reservoir for GBS. This may also increase the number of women given intrapartum prophylactic antibiotic treatment. However, the added clinical benefit of intrapartum and rectovaginal detection of GBS, compared to vaginal detection of GBS, to prevent EOGBS, is not yet clarified.

Based on the combined reference standard, the 14 PCR-negative but culture-positive samples appear counterbalanced by a higher number of PCR-positive samples from both PCR assays for the 19 culture-negative samples. This indicates that, for the detection of GBS in vaginal samples, there is no significant difference in sensitivity and specificity between PCR tests and culture. However, none of the three assays employed detected all GBS colonized women in this study.

Run time is critical because a rapid test result is needed in order to make a fast decision whether or not to administer prophylactic antibiotics before delivery in a childbirth unit. The difference in run time between the BD MAX and GenomEra PCR assays is only one hour (120 minutes vs. 60 minutes, respectively). Notably, the BD GeneOhm StrepB assay is a rapid and sensitive PCR assay (~ 1 hour) [13]. However, for optimal performance, the BD system still requires specially trained laboratory staff. With thorough training and maintenance of midwives’ qualifications, the GenomEra system is less complicated and more readily applicable as a point-of-care test in a clinical department than the BD MAX system. In few cases the intrapartum time can be less than 60 minutes and therefore administering prophylactic antibiotics to prevent infection has to rely on other information, e.g. risk factors. A future prospective study evaluating the capabilities of GenomEra GBS PCR on intrapartum fresh specimens performed on-site in a maternity ward is desirable in comparison with culture and/or another rapid and easy-to-use PCR assay.

## Conclusion

Both PCR assays performed comparably to culture of the intrapartum vaginal samples. In particular, the GenomEra GBS PCR assay is potentially as an easy and rapid on-site test for intrapartum detection of vaginal carriage of GBS at a maternity ward to identify women in labor who should receive intrapartum antibiotic prophylaxis.

## Supporting information

S1 Dataset Culture and PCR results. (XLSX)
